# Pokemon Silencing Leads to Bim-Mediated Anoikis of Human Hepatoma Cell QGY7703

**DOI:** 10.3390/ijms13055818

**Published:** 2012-05-15

**Authors:** Kun Liu, Feng Liu, Nannan Zhang, Shiying Liu, Yuyang Jiang

**Affiliations:** 1 Department of Chemistry, Tsinghua University, Beijing 100084, China; E-Mail: liukun09_lk@163.com; 2 The Ministry-Province Jointly Constructed Base for State Key Lab-Shenzhen Key Laboratory of Chemical Biology, the Graduate School at Shenzhen, Tsinghua University, Shenzhen 518055, China; E-Mails: xiaoee2728@126.com (N.Z.); liuleah@126.com (S.L.); 3 Department of Pharmacology and Pharmaceutical Sciences, School of Medicine, Tsinghua University, Beijing 100084, China

**Keywords:** Pokemon, anoikis, Bim, hepatoma

## Abstract

Pokemon is an important proto-oncogene that plays a critical role in cellular oncogenic transformation and tumorigenesis. Anoikis, which is regulated by Bim-mediated apoptosis, is critical to cancer cell invasion and metastasis. We investigated the role of Pokemon in anoikis, and our results show that Pokemon renders liver cells resistant to anoikis via suppression of Bim transcription. We knocked-down Pokemon in human hepatoma cells QGY7703 with small interfering RNAs (siRNA). Knockdown of Pokemon alone did not significantly affect the growth and survival of QGY7703 cells but notably enhanced their sensitivity to apoptotic stress due to the presence of chemical agents or cell detachment, thereby inducing anoikis, as evidenced by flow cytometry and caspase-3 activity assays. In contrast, ectopic expression of Pokemon in HL7702 cells led to resistance to anoikis. Dual-luciferase reporter and ChIP assays illustrated that Pokemon suppressed Bim transcription via direct binding to its promoter. Our results suggest that Pokemon prevents anoikis through the suppression of Bim expression, which facilitates tumor cell invasion and metastasis. This Pokemon-Bim pathway may be an effective target for therapeutic intervention for cancer.

## 1. Introduction

Pokemon, an erythroid myeloid ontogenic factor, is a member of the POK (POZ and Krüppel) family of transcriptional repressors [1]. Pokemon is a master regulator of B-cell and T-cell lymphoid fate and erythroid development and maturation [2,3]. However, aberrant over-expression of Pokemon in some human tumors, such as breast and liver cancer, lymphomas and adult malignant glioma [4–6], plays a key role in oncogenic transformation and tumorigenesis. Pokemon acts as a proto-oncogene by inhibiting the expression of several important tumor suppressors. For instance, Pokemon prevents apoptosis via suppression of p53 and Bim to promote cell survival with a tendency towards transformation. Pokemon suppresses p53 via the ARF-MDM2-p53 pathway but down-regulates Bim via direct binding to its promoter [7,8]. Pokemon also represses p21 and Rb expression to promote cell cycle progression. p21 is an inhibitor of cyclin-cdk2 complexes and a major regulator of the mammalian cell cycle [9–11], whereas Rb controls cell cycle progression at the G1/S checkpoint by suppressing the activity of an important transcription factor [12–14]. In addition, Pokemon promotes fatty acid synthesis to generate the membrane components phospholipids, which needed for rapid cell growth and proliferation [15,16]. Therefore, Pokemon has many functions in cancer cells that promote cell survival and cell cycle progression. These functions may also be essential for embryonic development. For example, the abrogation of Pokemon expression leads to lethal anemia in mouse embryos due to apoptosis of late-stage erythroblasts. This programmed cell death is mediated by upregulation of the pro-apoptotic factor, Bim, in Pokemon-null cells. GATA1 suppresses Bim-mediated apoptosis [3,17].

The Bcl-2 family of proteins is composed of both pro-apoptotic and anti-apoptotic members. The BH3-only protein Bim is pro-apoptotic, as it both activates Bax and suppresses the anti-apoptotic Bcl-2 proteins [18]. Bax is a key effector of mitochondria-mediated apoptosis, and its activity is conformationally regulated based on the ratio of BH3-only proteins, such as Bim, to the anti-apoptotic Bcl-2 family members [19,20]. Pro-survival Bcl-2 proteins are oncogenic; therefore, the loss of Bim expression and/or function may facilitate resistance to apoptotic stress during tumor development.

Cells rely on cell-cell and cell-matrix interactions for proper differentiation and survival [21,22]. Cells that lose these interactions die from programmed cell death through a process known as “anoikis” [23]. Anoikis relies heavily on the mitochondrial pathway of apoptosis in which Bim is a critical player that tips the balance of detached cells towards enhanced apoptotic potential. Bim transcription is found to be upregulated by sevenfold at 24 h after cell detachment. Knockdown of Bim renders NIH3T3, A549 and MCF10A cells able to survive anoikis [24–26]. Pokemon is a transcriptional suppressor of Bim [3]. Aberrantly over-expressed Pokemon may block apoptosis via suppression of detachment-induced Bim upregulation and thus render cells able to resist anoikis. Resistance to anoikis may allow cancer cells to survive during systemic circulation, thereby facilitating secondary tumor formation in distant organs [27,28]. Thus, the acquisition of anoikis resistance is essential for tumor cell invasion and metastasis. In this report, we studied the role of Pokemon in anoikis and identified a Pokemon-Bim-anoikis pathway.

## 2. Results and Discussion

### 2.1. Pokemon-Silencing Sensitizes Human Hepatoma to Anoikis and an Apoptosis Inducer

**Figure 1:**
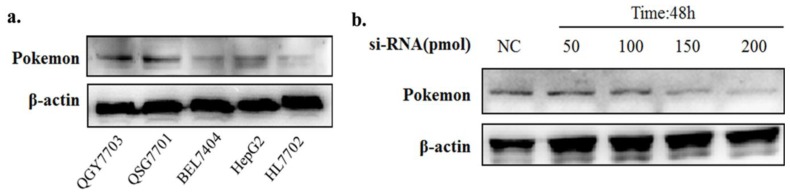
Pokemon expression level. (**a**) Pokemon protein in normal and cancer liver cells; (**b**) Dose-dependent siRNA silencing of Pokemon in QGY7703 cells.

We examined the expression of the Pokemon protein in three human hepatoma and two non-malignant liver cell lines. QGY7703, HepG2 and Bel7704 human hepatoma cells and QSG7701 and HL7702 human liver cells were studied. Pokemon expression is relatively higher in the three human hepatoma cells than in the two normal liver cell lines, particularly in human hepatoma QGY7703 cells compared to non-malignant liver HL7702 cells (Figure 1a).

**Figure 2:**
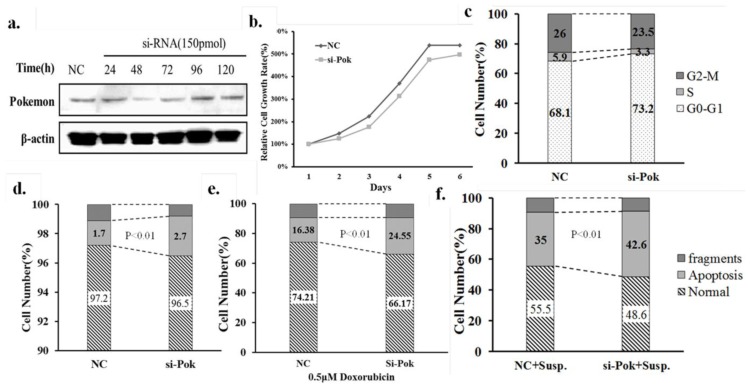
Sensitivity of QGY7703 cells to anoikis and Doxorubicin under Pokemon knockdown conditions. (**a**) Time course of Pokemon silencing by small interfering RNAs (siRNA) (150 pmol) in QGY7703 cells; (**b**) Cell growth rate; (**c**) FASC cell cycle analysis of QGY7703 cells treated with 150 pmol siRNA for 48 h after transfection; (**d**) FASC apoptosis analysis of QGY7703 cells treated with 150 pmol siRNA for 48 h; (**e**) FASC apoptosis analysis of QGY7703 cells treated with 150 pmol siRNA for 48 h, followed by 0.5 μM Doxorubicin treatment; (**f**) QGY7703 cells were treated with 150 pmol siRNA for 48 h and re-seeded on Ultra-Low attachment plates. After incubation for 24 h, cells were processed by FASC apoptosis analysis.

Silencing was optimized for the down regulation of Pokemon expression in QGY7703 cells using si-P1 which has been reported to effectively silence Pokemon in previous study [29]. Figure 1b shows the dose-dependent change in Pokemon expression after 48h si-P1 treatment. Figure 2a shows the time-dependent change in Pokemon expression with 150 pmol of si-P1. The data indicated that maximal Pokemon knockdown occurred at 48 h.

**Figure 3:**
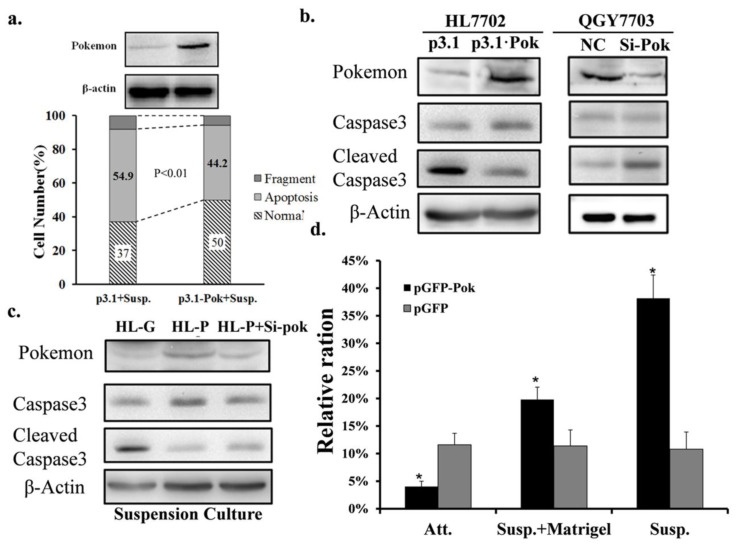
Anoikis resistance induced by ectopic expression of Pokemon in HL7702 cells. (**a**) HL7702 cells were treated with p3.1-Pok or p3.1 for 24 h, digested and re-plated on ultra-low attachment plates. After a 24 h incubation period, cells were processed by FASC apoptosis analysis. (**b**) Caspase-3 expression analysis. HL7702 cells were treated with pGFP or pGFP-Pok for 24 h and then treated as described above. QGY7703 cells were treated with 150 pmol siRNA for 48 h and then treated as described above. (**c**) HL-P, HL-G, and HL-P cells were treated with 150 pmol siRNA and then treated as described above. (**d**) HL7702 cells transfected with pGFP or pGFP-Pok were re-plated as described in the Materials and Methods. 48 h after re-seeding, the ratio of cells with GFP labeling to the total number of cells was calculated. The proportion of GFP alone or GFP-Pokemon cells subjected to regular, suspension or semi-suspension culture. ^*^ *p* < 0.01.

Pokemon is an important proto-oncogene; therefore, we are interested in the effect of Pokemon knockdown on QGY7703 liver cancer cells. We analyzed cell proliferation, cell cycle progression and apoptosis of QGY7703 cells when Pokemon is silenced. The results showed that Pokemon silencing led to a limited decrease in the cell growth rate (Figure 2b), a negligible cell cycle arrest at the G2/M checkpoint (Figure 2c), and a slight increase in apoptosis (Figure 2d) during regular culture. These changes were marginal with no statistical significance, indicating that under normal cultural conditions, Pokemon silencing does not significantly influence cell growth and survival. Furthermore, we tested the effect of Pokemon on the cellular response to diverse apoptotic stressors. Doxorubicin treatment (Aladdin, China) and detached culture conditions, which induces anoikis, were tested. The results showed that the 0.5 μM Doxorubicin treatment for 24 h triggered approximately 24.55% of the QGY7703 cells to undergo apoptosis when Pokemon is silenced, whereas 16.38% of cells treated with a scrambled siRNA were apoptotic (Figure 2e). Notably, under detached culture conditions, 42.6% of Pokemon-silenced QGY7703 cells underwent apoptosis compared to 35% of cells treated with a scrambled siRNA control (Figure 2f). The occurrence of anoikis was confirmed by determination of cleaved caspase-3 protein levels in Figure 3b.

### 2.2. Pokemon Enhances Resistance of HL7702 Cells to Anoikis

To further confirm the role of Pokemon in anoikis, we ectopically expressed it in human liver HL7702 cells, which have low endogenous expression of Pokemon. Figure 3a shows the Pokemon protein levels of HL7702 cells transfected with p3.1 or p3.1-Pok. As shown in Figure 3a, 44.2% of HL7702 cells that express Pokemon exogenously underwent apoptosis compared to 54.9% of the vector control cells when cultured under detachment conditions. This protective effect can also be detected in stable cell line HL-P and siRNA treated HL-P, as indicated by cleaved caspase-3 (Figure 3c).

The effect of Pokemon on anoikis was further confirmed by a cell counting study. In this study, green fluorescent protein (GFP)-tagged Pokemon was introduced into HL7702 cells. Following culture in detached, semi-detached or attached conditions for 48 h, viable fluorescent cells were counted using a fluorescent microscope. As shown in Figure 3d, the proportion of GFP-Pokemon-labeled cells was elevated by anoikis selection. These changes were not observed in vector control HL7702 cells. The results suggest that ectopic GFP-Pokemon expression protects HL7702 cells from anoikis.

### 2.3. Pokemon Prevents Anoikis through Suppression of Bim Expression

**Figure 4:**
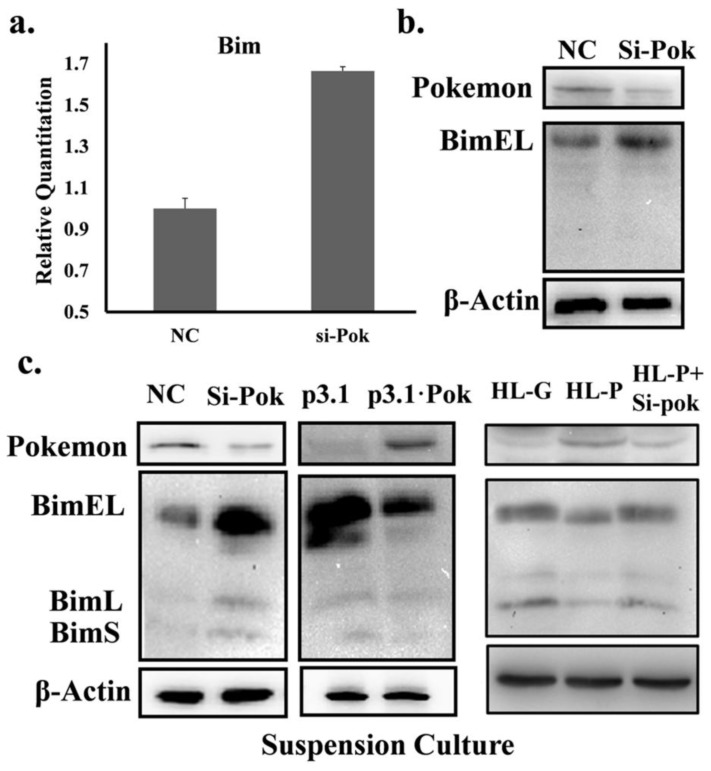
Pokemon suppresses detachment-induced Bim upregulation. (**a**) and (**b**) QGY7703 cells were treated with 150 pmol siRNA for 48 h and analyzed by a Bim Western blot or real-time RT-PCR; (**c**) QGY7703, HL-P, HL-G, and HL-P cells were treated with 150 pmol siRNA or scrambled RNA for 48 h and re-plated onto Ultra-Low attachment plates. After incubation for 24 h, Bim expression was assessed by Western blot. HL7702 cells were transfected with pGFP or pGFP-Pok. 24 h after transfection, Bim expression was analyzed as described above.

**Figure 5:**
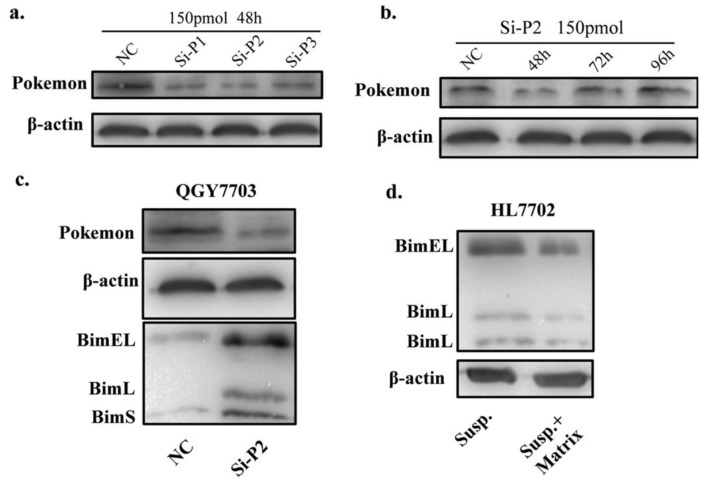
Pokemon silenced by si-P2. (**a**) Pokemon expression level of QGY7703 treated with scramble RNA, Si-P1, Si-P2, Si-P3 and scramle RNA; (**b**) Pokemon expression level of QGY7703 treated with Si-P2 for 48 h, 72hr and 96 h; (**c**) Bim expression level of QGY7703 after si-P2 treatment; (**d**) Bim expression level of HL7702 under suspension or semi-suspension culture.

Bim is an apoptotic protein that plays a significant role in anoikis [23], and the Pokemon-Bim pathway was reported in 2009 [3]. To understand the mechanism by which Pokemon suppresses anoikis, we further evaluated the effect of Pokemon on Bim expression. In attachment culture, Pokemon silencing did not appear to significantly alter Bim protein levels, although there was a clear increase in its mRNA levels (Figure 4a,b). Interestingly, in anoikis induced by detachment, Pokemon silencing significantly up-regulated Bim protein levels in QGY7703 cells, and in contrast, ectopic Pokemon expression significantly reduced Bim protein levels induced by anoikis in HL7702 cells (Figure 4c middle). We also detected Bim protein levels in HL-P cells that stably express Pokemon. The results showed that stably expressed Pokemon impaired detachment-induced Bim accumulation and anoikis and that this effect was reversed by siRNA knockdown of Pokemon (Figure 4c). Figure 5d shows that Matrigel weakened Bim accumulation. As in previous studies, the failure of Bim to accumulate corresponds with a failure to proceed with anoikis [24,25]. This finding also explains how Matrigel reverses the selective effect of anoikis in the fluorescence microscopy assay in which cells encounter less apoptosis stress in the presence of Matrigel; therefore, the survival advantage brought about by exogenous Pokemon would not be achieved. These results suggest that Pokemon regulates anoikis through suppression of Bim expression in QGY7703 and HL7702 cells.

To rule out the possibility of off-target effect, anoikis induced Bim accumulation was assessed using other si-RNAs. As shown in Figure 5a, si-P2 effectively silenced Pokemon and the time-course result was consistent with former ones (Figure 5b). Figure 5c shows that silencing of Pokemon by si-P2 also enhanced Bim accumulation under detachment. Thus, the increased Bim accumulation under detachment when Pokemon is silenced is not caused by off-target effects.

### 2.4. Pokemon Suppresses Bim Expression via Direct Binding to the Bim Promoter

**Figure 6:**
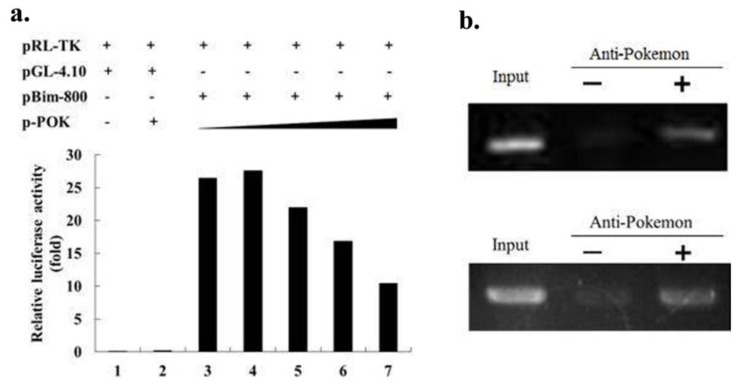
Pokemon suppresses Bim expression by directly binding to its promoter. (**a**) HL7702 cells were treated as indicated in the Materials and Methods. Forty-eight hour after transfection, the cells were processed by the dual luciferase reporter assay. p-POK is abbreviation for p3.1-POK. The doses of p-POK for each bar are 0, 0, 0, 0.1 μg, 0.2 μg, 0.4 μg, 0.8 μg from left to right. (**b**) Images showing ChIP PCR results from HepG2 (upper panel) and QGY7703 (lower panel) cells.

We further investigated the regulatory mechanism by which Pokemon, which is a transcription factor [3], affects Bim expression. Figure 4a shows that Pokemon silencing increased Bim mRNA levels, but Bim protein level was not significantly changed (Figure 4b). It seems that Pokemon silencing enhances Bim transcription, but the extra Bim protein is degradated so that the balance of Bcl-2 protein is maintained in cells. The resultsindicate that Pokemon may regulate Bim expression at the promoter level without influencing Bim degradation Data from a dual luciferase reporter assay confirmed the presence of a regulatory activity of Pokemon on the Bim promoter whose activity decreases along with Pokemon expression increasing (Figure 6a). A ChIP assay showed that Pokemon binds to the Bim promoter in both HepG2 and QGY7703 cells (Figure 6b). These data suggest that Pokemon suppresses Bim expression via direct binding to the Bim promoter.

### 2.5. Discussion

Liver cancer is the most prevalent and most lethal cancer worldwide, especially in Asia, where the incidence and mortality are strikingly high [30]. Pokemon has been reported to be highly expressed in some cancers, including hepatoma, and particularly in metastasized hepatoma [4]. These reports imply that there is a potential role for Pokemon in the development, progression, and especially metastatic transformation of hepatoma. This study revealed that Pokemon suppresses anoikis by inhibiting expression of the apoptotic protein Bim, thereby advancing the understanding of the pathological role of Pokemon in hepatoma.

The effect of Pokemon on cell growth and survival was marginal under normal culture conditions. Pokemon is a negative transcription factor that represses the tumor suppressor genes p53, p21 and Rb as well as the Bcl-2 family pro-apoptotic protein Bim [7,13]. All of these genes function in a homeostatic way under normal culture conditions but are induced or activated under certain circumstances, which lead to relevant biological consequences. Therefore, it is not surprising that Pokemon silencing did not have striking effects on cell growth and survival as shown by our results.

A question we asked in this study was whether Pokemon confers a survival advantage to cells in harsh environments, such as in the presence of chemical reagents and during cell detachment. Therefore, we tested the effect of Pokemon expression on QGY7703 cells exposed to a chemical inducer or cultured under detachment conditions. Either transient or stable expression or silencing of Pokemon affects the cell’s sensitivity not only to apoptosis triggered by a chemical inducer but also dramatically to anoikis. Solid evidence was also obtained in a fluorescence microscopy study. In this assay, the dramatically increased proportion of green cells after anoikis-selection and the correspondingly opposing effect of Matrigel supplements suggest a survival advantage for cells with increased Pokemon expression due to anoikis. These data suggest that Pokemon improves the ability of liver cancer cells to survive harsh environments caused by chemical reagent or detachment. This protective effect, which is essential for cells to survive in the presence of increased apoptotic stress, as is the case during the process of erythroid development, is exploited in tumor cells, as Pokemon allows QGY7703 cells to survive not only a higher concentration of Doxorubicin but also suspension culture. As a supplement to a previous study showing that Pokemon plays an important role in tumorigenesis, our study shows that Pokemon may also promote tumor cell invasion and metastasis by improving the ability of tumor cells to avoid anoikis because anoikis is a key barrier to metastasis for tumor cells.

Anoikis relies heavily on Bcl-2 family protein-mediated apoptosis. Bim is a key regulator of anoikis, and changes in Bim are sufficient to influence cell anoikis [19,26]. Our study showed that the Pokemon-Bim regulation pathway that exists in mice erythroid development also functions in the process of anoikis. Under regular culture conditions, Pokemon silencing in QGY7703 cells had a limited effect on Bim protein levels, although its mRNA levels were increased. However, under detachment stress, Pokemon greatly changed Bim protein expression. In the cell lines we studied, increased Pokemon levels were correlated with decreased Bim accumulation. Studies of the Bim promoter using luciferase reporter and ChIP assays further revealed that Pokemon regulates anoikis in liver cells by controlling Bim expression via direct binding to the Bim promoter. We proposed a hypothetical pathway in which Pokemon regulates anoikis. When cells with low Pokemon levels are exposed to detachment conditions, Bim transcription is upregulated and Bim protein accumulates; therefore, the cells undergo apoptosis. When Pokemon is overexpressed in cells, Bim transcription is blocked, which leads to a failure to accumulate Bim protein, causing an impairment of apoptosis induced by detachment. It is noteworthy that under normal culture conditions (non-anoikis), Pokemon silencing enhanced the mRNA but not the protein levels of Bim, which indicates the possibility that a translational regulatory mechanism exists. Further study is warranted to test this hypothesis.

## 3. Experimental Section

### 3.1. Cell Lines and Culture

Human hepatoma (QGY7703, HepG2, and BEL7404) and non-malignant QSG7701 and HL7702 liver cell lines were purchased from the Shanghai Institutes of Biological Sciences Cell Bank (Shanghai, China). QGY7703 and HepG2 cells were maintained in Dulbecco’s Modified Eagle medium (DMEM) containing 10% fetal bovine serum (FBS) at 37 °C, 5% CO_2_. QSG7701, BEL7404 and HL7702 cells were maintained in RPMI 1640 medium with 10% FBS. Stable cell lines were established as previously described [31]. Briefly, HL7702 cells were transfected with the corresponding plasmids. After 48 h, the cells were trypsinized, diluted, and re-seeded onto plates, followed by G418 selection for stable transfectants. For detachment culture, cells were seeded in Ultra-Low attachment 6-well plates (Corning, Glendale, Arizona, USA), and 1% methylcellulose was added to full media to avoid the survival effect caused by cell clumping.

### 3.2. Plasmid Construction

PCR-amplified Pokemon cDNA was cloned into the pcDNA3.1(−) and pEGFP-N3 vectors, and the resulting constructs were named p3.1-POK and pGFP-POK, respectively [32]. The Bim promoter was cloned into the pGL4.10 vector and named p4.10-bim800. All vectors were constructed according to standard cloning procedures. The primers used for the Bim promoter were Bim-p forward: 5′-ATA GGT ACC ATG CCT CCC GCC CTC ACC CGG GA-3′, and Bim-p reverse: 5′-TTA CTC GAG TTG AGC TCC AAC AAA CTG CAG ACC-3′.

### 3.3. Pokemon Silencing by siRNA

Three siRNAs were synthesised and were used to target Pokemon as described in previous studies [31]. The sequences are as follows: si-P1 sense 5′-GCU GGA CCU UGU AGA UCA Att-3′, si-P2 sense 5′-UCA AGA AAG ACG GCU GCA Att-3′ and si-P3 sense5′-GCA GAU GAU GUC AUC GGU Gtt-3′. For siRNA delivery, cells (2 × 105 in Opti-MEM I media) were mixed gently with siRNA and OligofectAMINE (Invitrogen, Grand Island, New York, USA) in a final volume of 0.5–1.5 mL, according the manufacturer’s instructions, and incubated at 37 °C, 5% CO_2_ for 4 h, followed by the addition of an equal volume of fresh media containing 20% FBS. Cells were continuously incubated until harvest.

### 3.4. Flow Cytometry Analysis

For apoptosis analyses, cells were harvested and stained using an Annexin V-FITC apoptosis analysis kit (Beyotime, China) according to the manufacturer’s instructions. For cell cycle analyses, the cells were trypsinized, washed with PBS twice, and fixed with ethanol (−20 °C). Before flow cytometry analyses, the ethanol was removed, and 1 mL of freshly made dying solution (0.05 mg/mL PI, 0.025 mg/mL RNase, 0.0025% TritonX-100) was added. After staining for 5 min, the cells were subjected to flow cytometry analysis, using a MoFloTM XDP cytometer (Beckman Coulter, Atlanta, Georgia, USA). Three replicates were performed.

### 3.5. Real-Time PCR

Total RNA was extracted using the Trizol reagent (Invitrogen, Grand Island, New York, USA) according to the manufacturer’s instructions and quantified with a Nano-drop ND-1000 (Thermo Scientific, Wilmington, Delaware, USA). The quality of the RNA was verified by agarose-gel electrophoresis. Real-time PCR was performed on an AB 7500 Real Time PCR System using the cDNA obtained above. The results were analyzed by the AB 7500 PCR system SDS software (Applied Biosystems, Bedford, Ohio, USA). Three replicates were performed.

### 3.6. Western Blot

Lysates (30 μg protein) of treated or non-treated cells were resolved by SDS-PAGE, transferred to PVDF membranes, and blocked with 0.5% skim milk in 1× TBS. Membranes were incubated with primary antibodies overnight at −4 °C and washed three times for 10 min each in TBST (20 mM Tris-HCl, pH 7.5, 150 mM NaCl, 0.05% Tween-20). The membranes were then incubated with HRP-conjugated secondary antibodies for 1.5 h at room temperature. Images were acquired using the GeL Doc EQ image reader system (Bio-Rad, Munich, Germany). Protein amounts were normalized according to beta-actin monoclonal antibody staining. The antibodies used included anti-human Pokemon (Sigma, USA), anti-human BIM, anti-caspase3 and anti-cleaved caspase-3 (Cell Signaling Technology, Danvers, Massachusetts, USA), and anti-human beta-actin (Beyotime, DaLian, China).

### 3.7. Chromatin Immunoprecipitation

Subconfluent QGY7703 or HepG2 cells were fixed with 1% formaldehyde, washed with PBS, lysed with SDS lysis buffer (50 mM Tris-HCl, pH 8.1, 5 mM EDTA, 0.1% deoxycholate, 1% Triton X-100,150 mM NaCl, 1% protease inhibitor cocktail), and sonicated to generate DNA fragments of 500–1000 bp in size. Pre-immune serum was added to the sonicated supernatant and incubated for 1 h at 4 °C with rotation. Protein G Dynabeads (Invitrogen, USA) were added and incubated for 1 h at 4 °C. A magnetic separator was used to collect the beads. The supernatant was diluted 2-fold with ChIP dilution buffer (0.01% SDS, 1% Triton X-100, 2 mM Tris-HCl, pH 8.1, and 150 mM NaCl) and incubated with either anti-Pokemon antibody or IgG as a control at 4 °C overnight. Protein G Dynabeads were added to the mixture, incubated for 2 h at 4 °C with rotation, and separated with the magnetic separator. DNA-Pokemon-antibody complex was eluted with TE buffer at pH 3.0. After EDTA and proteinase K treatment, DNA was extracted with phenol/chloroform and precipitated with ethanol. The primers used were Bim1-forward: 5′-TCG CGA GGA CCA ACC CAG TC-3′ and Bim1-reverse: 5′-CCG CTC CTA CGC CCA ATC AC-3′.

### 3.8. Dual Luciferase Reporter Assay

A dual luciferase reporter assay system was purchased from Promega, USA and used according to the manufacturer’s instructions. Briefly, HL7702 cells were cultured in RPMI 1640 medium with 10% FBS on 6-well plate and were transfected with 0.8 μg of pRL-TK, 0.8 μg of pGL-4.10 or pBim-800 and 0.8μg of pcDNA3.1 and p3.1-POK using FuGENE HD (Roche, USA). After 36 h of incubation, cells were harvested, lysed, and 10 μL of the resulting lysate was used to test luciferase activity. Different Pokemon expression levels are obtained by transfecting different dose of p3.1-POK.

### 3.9. Cell Counting Assay

HL7702 cells were transfected with pGFP-POK or control vector for 24 h, trypsinized and divided into three equal parts, labeled I, II and III. Part I was re-seeded onto regular six-well plates. Parts II and III were plated on Ultra-Low attachment 6-well plates. Five percent matrigel was added to plates containing Part II cells. After 48 h, green fluorescent cells were counted in five random areas of view using a fluorescence microscope (Olympus, Japan). Three replicates were performed.

## 4. Conclusions

Pokemon render liver cells better ability to resist anoikis by suppressing Bim accumulation via direct bind to Bim promoter. The Pokemon-Bim pathway is essential in mice erythroid development [3], but these new research findings reveal a novel function of this pathway in tumor progression, providing experimental support for the potential role of Pokemon in tumor metastasis. Pokemon-targeted cancer therapy may effectively suppress not only carcinoma in situ but also tumor metastasis and replacement.
